# HPV co-factors related to the development of cervical cancer: results from a population-based study in Costa Rica

**DOI:** 10.1054/bjoc.2001.1779

**Published:** 2001-05

**Authors:** A Hildesheim, R Herrero, P E Castle, S Wacholder, M C Bratti, M E Sherman, A T Lorincz, R D Burk, J Morales, A C Rodriguez, K Helgesen, M Alfaro, M Hutchinson, I Balmaceda, M Greenberg, M Schiffman

**Affiliations:** Division of Cancer Epidemiology and Genetics, National Cancer Institute, Bethesda, MD; Caja Costarricense de Seguro Social, San Jose, Costa Rica; Department of Pathology, The Johns Hopkins Medical Institutions, Baltimore, MD; Digene Corporation, Silver Spring, MD; Albert Einstein College of Medicine, Bronx, NY; Information Management Services, Silver Spring, MD; Womens and Infants' Hospital, Providence, RI; 8OMNIA, Blue Bell, PA

**Keywords:** cervix, human papillomavirus, cancer, smoking, parity

## Abstract

We examined factors associated with high-grade squamous intraepithelial lesions (HSIL) and cervical cancer among human papillomavirus (HPV)-infected women in a prevalent case–control study conducted within a population-based cohort of 10 077 women in Costa Rica. We compared 146 women with HPV-positive HSIL or cancer (HSIL/CA) against 843 HPV-positive women without evidence of HSIL/CA. Subjects completed a risk factor questionnaire. We evaluated the associations between exposures and HSIL/CA among women positive for any HPV and restricted to those positive for high-risk HPV types. Risk of HSIL/CA increased with increasing number of live births (*P*_trend_= 0.04). Women who smoked 6+ cigarettes/day had a RR for HSIL/CA of 2.7 (95% CI = 1.1–6.7) compared to non-smokers. Current use of barrier contraceptives was associated with a reduction in risk of HSIL/CA (RR = 0.39; 95% CI = 0.16–0.96). Sexual behaviour and a self-reported history of sexually transmitted diseases (STDs) other than HPV were not associated with HSIL/CA. Oral contraceptive use was associated with HSIL/CA among women with <3 pregnancies. Effects were similar in analysis restricted to women positive for high-risk HPV types. Among women positive for high-risk HPV types, 44% of HSIL/CA could be attributed to multiparity (≥3 pregnancies) and/or smoking. Among HPV-positive women, multiparity and smoking are risk factors for HSIL/CA. Oral contraceptive use may be associated with HSIL/CA in subgroups of women. © 2001 Cancer Research Campaign http://www.bjcancer.com


British Journal of Cancer (2001) 84(9), 1219–1226
© 2001 Cancer Research Campaign
doi: 10.1054/ bjoc.2001.1779, available online at http://www.idealibrary.com on IDEAL®

http://www.bjcancer.com

### HPV co-factors related to the development of cervical cancer: results from a population-based study in Costa Rica

**A Hildesheim[1], R Herrero[2], PE Castle[1], S Wacholder[1], MC Bratti[2], ME Sherman[1,3], AT Lorincz[4], RD Burk[5], J Morales[2], AC Rodriguez[2], K Helgesen[6], M Alfaro[2], M Hutchinson[7], I Balmaceda[2], M Greenberg[8] and M Schiffman[1]**

[1]Division of Cancer Epidemiology and Genetics, National Cancer Institute, Bethesda, MD; [2]Caja Costarricense de Seguro Social, San Jose, Costa Rica; [3]Department of Pathology, The Johns Hopkins Medical Institutions, Baltimore, MD; [4]Digene Corporation, Silver Spring, MD; [5]Albert Einstein College of Medicine, Bronx, NY; [6]Information Management Services, Silver Spring, MD; [7]Womens and Infants' Hospital, Providence, RI; [8]OMNIA, Blue Bell, PA

**Summary** We examined factors associated with high-grade squamous intraepithelial lesions (HSIL) and cervical cancer among human papillomavirus (HPV)-infected women in a prevalent case–control study conducted within a population-based cohort of 10 077 women in Costa Rica. We compared 146 women with HPV-positive HSIL or cancer (HSIL/CA) against 843 HPV-positive women without evidence of HSIL/CA. Subjects completed a risk factor questionnaire. We evaluated the associations between exposures and HSIL/CA among women positive for any HPV and restricted to those positive for high-risk HPV types. Risk of HSIL/CA increased with increasing number of live births ($P_{trend}$ = 0.04). Women who smoked 6+ cigarettes/day had a RR for HSIL/CA of 2.7 (95% CI = 1.1–6.7) compared to non-smokers. Current use of barrier contraceptives was associated with a reduction in risk of HSIL/CA (RR = 0.39; 95% CI = 0.16–0.96). Sexual behaviour and a self-reported history of sexually transmitted diseases (STDs) other than HPV were not associated with HSIL/CA. Oral contraceptive use was associated with HSIL/CA among women with <3 pregnancies. Effects were similar in analysis restricted to women positive for high-risk HPV types. Among women positive for high-risk HPV types, 44% of HSIL/CA could be attributed to multiparity (≥3 pregnancies) and/or smoking. Among HPV-positive women, multiparity and smoking are risk factors for HSIL/CA. Oral contraceptive use may be associated with HSIL/CA in subgroups of women. © 2001 Cancer Research Campaign http://www.bjcancer.com

**Keywords**: cervix; human papillomavirus; cancer; smoking; parity

The vast majority of cervical cancer cases are attributable to human papillomavirus (HPV) infection (Bosch et al, 1995; Schiffman et al, 1996; Nobbenhuis et al, 1999; Herrero et al, 2000). Our current challenge is to identify factors involved in the rare progression of HPV infection, which is common and usually benign, to cervical cancer and its immediate precursor, high-grade squamous intraepithelial lesions (HSIL).

Results from studies of HPV 'co-factors' suggest that reproductive factors, contraceptive use, cigarette smoking, and correlates of sexual behaviour other than HPV infection might be associated with HSIL and cervical cancer (henceforth referred to as HSIL/CA) (Bosch et al, 1992; Munoz et al, 1993; Becker et al, 1994; Moreno et al, 1995; Kjaer et al, 1996; Chaouki et al, 1998; Chichareon et al, 1998; Ho et al, 1998; Kruger-Kjaer et al, 1998; Olsen et al, 1998; Ngelangel et al, 1998; Roteli-Martins et al, 1998). In most of these studies, HPV infection was accounted for through statistical adjustment, i.e. by averaging the impact of other factors in HPV-negative and HPV-positive cases and controls. Given strong association between HPV and HSIL/CA (relative risks of ≥ 50), it is unclear whether effects observed for co-factors (relative risks usually < 3) in these previous studies are real or due to residual confounding by HPV.

*Received 23 November 2000*
*Revised 19 February 2001*
*Accepted 19 February 2001*

*Correspondence to:* A Hildesheim

To address this concern, we conducted a prevalent case–control study within a 10 077 woman population-based study in Guanacaste, Costa Rica. We evaluated factors associated with progression of HPV infection and its early cytologic manifestation (LSIL) by comparing 146 HPV-positive cases of HSIL/CA against a group of 843 HPV-positive cohort members who did not have concurrent HSIL/CA. We chose our control group to include only non-cases (not diagnosed with HSIL) that were truly at risk of becoming a case (infected with HPV). Although this study design likely attenuated the associations of cofactors that are associated with the acquisition and possible persistence of infection, it avoided potential residual confounding by HPV. Thus, by including only HPV-infected women in a strictly population-based study, we were better able to estimate properly the role of possible HPV co-factors.

### METHODS

### Cohort base

A population-based cohort was established in Guanacaste, Costa Rica in 1993/4 (Herrero et al, 1997, 2000). The study was conducted after approval by the NCI and local institutional review boards, and all participants provided informed consent. Cluster sampling was utilized to select a representative sample of the adult female population of Guanacaste (*n* = 10 738 eligible women). 10 049 women (94% of eligibles) agreed to visit one of our study clinics. Because few women were expected to have screen-detected invasive cancer, we supplemented our sample by including women from Guanacaste diagnosed with incident cervical cancer in 1993/4. We attempted to recruit all cervical cancer cases from major centres to which Guanacaste residents are referred for diagnosis and treatment (Herrero et al, 1997). 28 of 31 additional cervical cancer cases identified in this manner were alive and participated. The total number of women in our cohort was therefore 10 077.

### Data and specimen collection

Participants responded to a risk factor questionnaire that assessed information on socio-demographic characteristics; sexual, reproductive, and birth control practices; cigarette smoking; and sexually transmitted diseases (STDs). Sexually active women underwent a pelvic examination, at which time a Pap smear was prepared, cells were collected for semi-automated ThinPrep cytology (Cytyc Corp, Boxborough, MA), Cervigrams (National Testing Laboratories, Fenton, MO) were taken, and cervical cells were collected for HPV DNA testing (Herrero et al, 1997, 2000). 583 virgins, 291 women who refused to have a pelvic exam or for whom physical problems prevented a pelvic, and 621 women who reported having a hysterectomy were excluded. Thus, 8 582 women were considered for the present study (Figure 1).

Women with cervical abnormalities by visual inspection, cytology or cervicography, or who were in a 2% random sample of the population were referred to colposcopy, at which time a second questionnaire obtained medical history information and information on douching practices.

Lesions visible at colposcopy were biopsied. Based on review of cytology, cervigram and histology, each woman was assigned a diagnosis (Herrero et al, 1997; Schiffman et al, 1999). 40 women (including supplemental cases) were diagnosed with cervical cancer (39 histologically confirmed and one surgically evident), 128 with HSIL (93% histologically confirmed as having cervical intraepithelial neoplasia 2 or 3 (CIN2/3); the remainder confirmed by at least 2 reviewed cytologic diagnoses), and 189 with LSIL (39% histologically confirmed as having cervical intraepithelial neoplasia 1 or condylomatous atypia and the remainder confirmed as LSIL by at least 2 screening tests). The remaining 8225 participants had equivocal lesions ($n$ = 661) or were cytologically normal ($n$ = 7564).

### HPV DNA testing

Cervical cells were tested for HPV DNA using the Hybrid Capture Tube test (Cox et al, 1995). This assay detects infection by 16 HPV types (6, 11, 16, 18, 31, 33, 35, 39, 42, 43, 44, 45, 51, 52, 56, 58). Testing was performed for 8563 (99.8%) of the 8582 eligible women with a pelvic examination. The PCR-based L1 consensus primer HPV test was also performed on a set of 2974 women (Hildesheim et al, 1994; Herrero et al, 2000). For the present study, we utilized PCR data from 2300 women selected for PCR testing due to 1) an abnormal screening test (1702), 2) HPV positivity by Hybrid Capture (303), or 3) selection as a random sample of the remaining cohort (295). PCR results from a group of women ($n$ = 674) selected for testing on the basis of their sexual behaviour were not utilized (except those that were selected as part of the random sample of the remaining cohort) to avoid biasing our analysis of co-factors. PCR testing detected type-specific infection by 44 HPV types (2, 6, 11, 13, 16, 18, 26, 31, 32, 33, 34, 35, 39, 40, 42, 45, 51, 52, 53, 54, 55, 56, 57, 58, 59, 61, 62, 64, 66, 67, 68, 69, 70, 72, 73, 83, AE2, AE4, AE5, AE6, AE7, AE8, W13B, PAP155) and infection with uncharacterized types (Herrero et al, 2000). Using Hybrid Capture and PCR HPV testing results, women were classified as 1) positive for high-risk HPV types (16, 18, 31, 33, 35, 39, 45, 51, 52, 56, 58, 59, 68) if they tested positive for these types by either method, 2) negative for high-risk HPV types but positive for low-risk HPV types (all other HPV types and uncharacterized types) if they tested negative for high-risk types by both Hybrid Capture and PCR (when available) and positive for low-risk types by either method, or 3) HPV DNA-negative by Hybrid Capture or both HPV DNA assays. By these criteria, 760 women were positive for high-risk HPV types, 229 were positive for low-risk types only, and 7574 were negative. Among positives, 596 (60.3%) were positive by Hybrid Capture and PCR, 24 (2.4%) were positive by Hybrid Capture and had missing PCR data, 129 (13.0%) were positive by Hybrid Capture but negative by PCR, and 240 (24.3%) were negative by Hybrid Capture but positive by PCR, confirming the higher sensitivity of PCR.

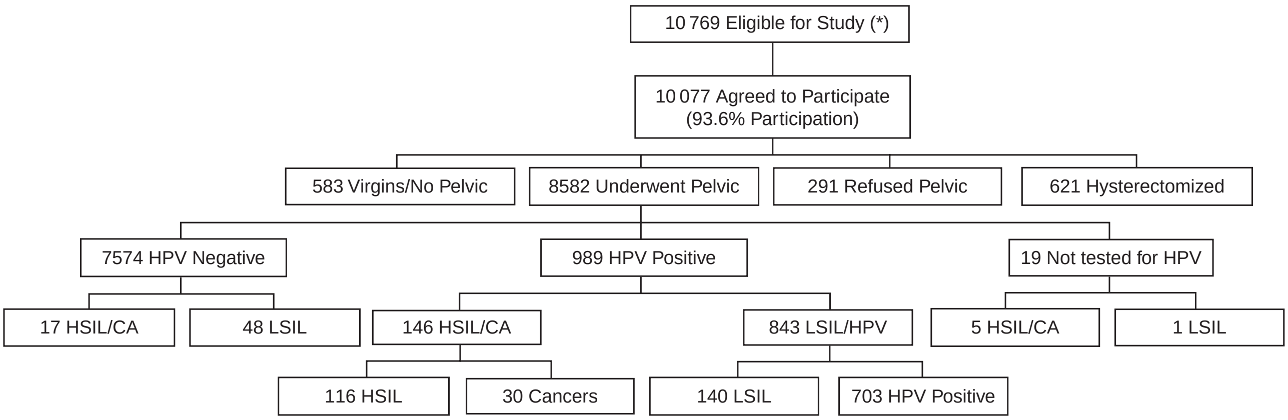

* Includes 10 738 women selected as part of our cohort and 31 supplemental invasive cancer cases (see text)

**Figure 1** Summary of study subject selection

*© 2001 Cancer Research Campaign*

### Case–control study subjects

Cases were HPV-positive women diagnosed with prevalent HSIL/CA at entry into our cohort study ($n$ = 146). These cases comprised 86.9% (146/168) of all HSIL/CA cases in our cohort. Of the 22 potential cases who were excluded, 17 (10.1%) tested negative for HPV and 5 (3.0%) were not tested. HSIL/CA cases were combined because HSIL is recognized as the immediate precursor to cervical cancer. Although cancer cases were older than HSIL cases (median age = 47 years vs 34 years, respectively), comparison of HSIL ($n$ = 116) and cancer ($n$ = 30) cases controlling for age revealed no notable differences between the two groups with respect to the many risk factors examined.

HPV-positive women with and without a cytological diagnosis of LSIL at entry into our cohort were included in a single control group ($n$ = 843) since LSIL is understood to be the cytological manifestation of HPV infection (Schiffman and Brinton, 1995). The 843 controls included 140 HPV DNA-positive women with LSIL and 703 women with HPV DNA positivity only. Those with LSIL comprised 74.1% (140/189) of all LSILs in our cohort. Of the 49 potential controls who were excluded, 48 (25.4%) tested negative for HPV and one (0.5%) was not tested. Women with LSIL were slightly younger than those without (median age = 29 years for the LSIL vs 32 years for the HPV positive only group). After control for age, the only notable differences observed between controls with and without LSIL were a higher likelihood of those with LSIL to test positive for high-risk HPV types (age-adjusted RR = 2.0; 95% CI = 1.2–3.3) and a marginally greater likelihood of these women to have had sexual intercourse >1/week in the past year (age-adjusted RR = 1.5; 95% CI = 1.0–2.3).

### Statistical analysis

The relative risk (RR), as estimated by the odds ratio, was the measure of association between exposures and disease. 95% confidence intervals (CI) determined the statistical significance of findings. Logistic regression analysis permitted adjustment for age and confounding co-factors (Breslow and Day, 1980). Dose–response relationships were tested for statistical significance by treating the categorical variable as continuous and evaluating whether the resultant beta coefficient departed from zero. The fraction of HSIL/CA among women infected with high-risk HPVs that is attributable to exposure to co-factors was estimated by the method of Benichou (Benichou, 2000).

Analysis was conducted among all HPV-positive subjects and also restricted to women positive for high-risk HPV types (136 cases and 624 controls). Both sets of results are presented in the tables. HSIL cases comprise women with CIN2 and CIN3. To alleviate concerns that CIN2 cases might be false positive cases (i.e. LSILs rather than HSILs) or that they may represent a different biological entity, analyses were also repeated after exclusion of CIN2 cases. While CIN2 cases were younger than CIN3/cancer cases (median age = 31 and 38, respectively), results from the analysis restricted to cases with CIN3 or cancer yielded similar results (data not shown). The few differences noted in this restricted analysis are presented in the text. Analyses adjusted for age (18–24, 25–29, 30–44, 45–64, 65+), HPV type (high-risk vs low-risk), number of pregnancies (0/1, 2/3, 4/5, 6+), and number of cigarettes smoked per day (0, 1–5, 6+) are presented. Finer adjustments for age did not significantly alter the estimates. Other factors did not confound associations presented.

Because controls were defined based on prevalent infection, there was concern that risk estimates for cofactors of interest could be biased by the overrepresentation in our control group of women with either persistent viral infection or women with recently acquired infections. We therefore conducted a parallel analysis comparing cases to all cohort non-case members in our population-based study, statistically adjusting for HPV. Results of this analysis were identical to the results presented and therefore are not shown.

### RESULTS

HSIL/CA cases were, on average, 4 years older than controls (median age = 36 for HSIL/CA and 32 for LSIL/HPV). After controlling for age, no association was seen between level of education and risk of HSIL/CA (data not shown). The Pap smear screening behaviour of cases and controls was very similar; 85.6% of cases and 82.8% of controls reported ever having had a smear and no association was observed between the interval since last smear or the number of smears and HSIL/CA (age-adjusted RR for 5+ Paps compared to none = 1.1; 95% CI = 0.62–2.1). The age-adjusted risk of HSIL/CA was 5.2 times higher (95% CI = 2.8–10.1) in women with high-risk HPV infection than those with low-risk types. Only 10 HSIL/CA cases were positive for low-risk HPV types alone, 5 of whom were positive for uncharacterized types (Herrero et al, 2000).

Age at first intercourse, interval between menarche and sexual debut, and total number of sexual partners were not associated with disease (Table 1). There was a suggestion that number of sexual partners in the preceding year was negatively associated with HSIL/CA ($P_{trend}$ = 0.14) and that an increased frequency of sexual intercourse was associated with HSIL/CA ($P_{trend}$ = 0.15). Women who reported ever being pregnant were at increased risk of disease (RR = 4.5, 95% CI = 1.1–19), and among ever-pregnant women risk increased with increasing number of pregnancies ($P_{trend}$ = 0.10) and with live births ($P_{trend}$ = 0.04). Similar trends were observed with the number of full-term ($P_{trend}$ = 0.06) and with number of vaginal births ($P_{trend}$ = 0.05) (data not shown). Having had one miscarriage/abortion (RR = 0.71, 95% CI = 0.42–1.2) or 2 or more miscarriages/abortions (RR = 0.60, 95% CI = 0.31–1.2) was not significantly associated with risk of HSIL/CA.

No significant associations were observed for age at first pregnancy for women who had their first pregnancy between the ages 20–24 (RR = 1.2, 95% CI = 0.76–1.2) and 25+ (RR = 0.77, 95% CI = 0.37–1.6) compared to women who had their first pregnancy when they were under 20 years of age. No significant association was observed with any occurrence of stillbirths (RR = 0.53, 95% CI = 0.23–1.2) or tubal/ectopic pregnancies (RR = 1.9, 95% CI = 0.37–10). No significant association were observed for women who had one caesarean section (RR = 1.2; 95% CI = 0.61–2.4) or 2+ caesarean sections (RR = 0.70; 95% CI = 0.29–1.7).

Overall, no significant association was observed between oral contraceptive use and risk of HSIL/CA (Table 2). However, a significant association between oral contraceptives and HSIL/CA was evident among women with <3 pregnancies ($P_{trend}$ = 0.02; Table 3), an effect that persisted after further adjustment for barrier contraceptive use. Current use of barrier contraceptives (condoms or diaphragms) was associated with a reduced risk of HSIL/CA; further adjustment for oral contraceptive use did not affect the pattern observed. Use of intrauterine devices and tubal ligations were not related to risk (data not shown). There was a suggestion

© 2001 Cancer Research Campaign 

**Table 1** Distribution and risk of HSIL/CA associated with sexual and reproductive practices

| | Overall analysis | | | | Analysis restricted to women positive for high risk HPV types[b] | |
|---|---|---|---|---|---|---|
| **Variable**[a] | **% Cases ($n$ = 146)** | **% Controls ($n$ = 843)** | **RR**[c] | **95% CI** | **RR**[d] | **95% CI** |
| Age at first intercourse | | | | | | |
| <16 | 26.7 | 24.3 | 1.0 | | 1.0 | |
| 16–19 | 49.3 | 52.5 | 1.1 | [0.69–1.7] | 1.2 | [0.72–1.9] |
| 20+ | 24.0 | 23.2 | 1.2 | [0.69–2.1] | 1.2 | [0.65–2.1] |
| Years between menarche and sexual debut | | | | | | |
| <2 | 14.4 | 14.6 | 1.0 | | 1.0 | |
| 2–3 | 34.9 | 27.2 | 1.3 | [0.72–2.3] | 1.3 | [0.69–2.3] |
| 4–5 | 25.3 | 25.2 | 1.3 | [0.69–2.4] | 1.3 | [0.67–2.4] |
| 6+ | 25.4 | 33.0 | 0.93 | [0.50–1.7] | 0.86 | [0.45–1.6] |
| Number of sexual partners in lifetime | | | | | | |
| 1 | 34.9 | 41.5 | 1.0 | | 1.0 | |
| 2–3 | 43.2 | 41.7 | 1.2 | [0.76–1.8] | 1.2 | [0.78–1.9] |
| 4+ | 21.9 | 16.8 | 1.1 | [0.67–1.9] | 1.0 | [0.59–1.8] |
| Number of sexual partners in past year | | | | | | |
| 0 | 22.6 | 17.3 | 1.0 | | 1.0 | |
| 1 | 76.0 | 77.9 | 0.87 | [0.52–1.4] | 0.89 | [0.53–1.5] |
| 2+ | 1.4 | 4.8 | 0.23 | [0.05–1.1] | 0.24 | [0.05–1.1] |
| | | | $P_{trend}$ = 0.14 | | $P_{trend}$ = 0.16 | |
| Frequency of sexual intercourse in past year[e] | | | | | | |
| <1/week | 21.2 | 27.0 | 1.0 | | 1.0 | |
| 1/week | 26.6 | 22.6 | 1.5 | [0.83–2.8] | 1.7 | [0.91–3.2] |
| >1 – ≤2/week | 21.2 | 24.1 | 1.3 | [0.67–2.5] | 1.4 | [0.70–2.7] |
| >2–<4/week | 20.4 | 17.7 | 1.6 | [0.81–3.1] | 1.3 | [0.66–2.7] |
| 4+/week | 10.6 | 8.6 | 1.8 | [0.82–4.1] | 2.1 | [0.90–4.7] |
| | | | $P_{trend}$ = 0.15 | | $P_{trend}$ = 0.23 | |
| Ever pregnant | | | | | | |
| No | 1.4 | 7.8 | 1.0 | | 1.0 | |
| Yes | 98.6 | 92.2 | 4.5 | [1.1–19] | 4.6 | [1.1–20] |
| # Pregnancies | | | | | | |
| 1 | 9.7 | 19.4 | 1.0 | | 1.0 | |
| 2 | 11.1 | 20.1 | 0.97 | [0.44–2.1] | 0.86 | [0.38–1.9] |
| 3 | 16.7 | 14.9 | 1.7 | [0.78–3.6] | 1.4 | [0.65–3.2] |
| 4–5 | 25.7 | 17.1 | 2.3 | [1.1–4.8] | 2.3 | [1.1–4.9] |
| 6–8 | 22.9 | 13.1 | 2.7 | [1.2–5.9] | 2.5 | [1.1–5.6] |
| 9+ | 13.9 | 15.3 | 1.1 | [0.47–2.7] | 1.1 | [0.43–2.6] |
| | | | $P_{trend}$ =0.10 | | $P_{trend}$ =0.12 | |
| # Live births[f] | | | | | | |
| 0–1 | 11.1 | 23.3 | 1.0 | | 1.0 | |
| 2 | 11.8 | 20.1 | 1.2 | [0.56–2.5] | 1.0 | [0.48–2.2] |
| 3 | 18.7 | 16.7 | 1.7 | [0.86–3.6] | 1.5 | [0.73–3.2] |
| 4–5 | 29.9 | 15.4 | 3.7 | [1.8–7.4] | 3.5 | [1.7–7.2] |
| 6–8 | 16.0 | 12.9 | 2.2 | [1.0–5.0] | 2.2 | [0.98–5.0] |
| 9+ | 12.5 | 11.6 | 1.5 | [0.61–3.5] | 1.4 | [0.56–3.4] |
| | | | $P_{trend}$ =0.04 | | $P_{trend}$ =0.04 | |

[a]1 control missing age at 1st intercourse information. 3 controls missing information on years between menarche and sexual debut. 1 control missing information on number of partners in the past year. 1 control missing information on frequency of sexual intercourse in past year. [b]High-risk HPV types include types 16, 18, 31, 33, 35, 39, 45, 51, 52, 56, 58, 59, 68. [c]RR adjusted for age, HPV type, number of pregnancies, and number of cigarettes/day. [d]RR adjusted for age, number of pregnancies, and number of cigarettes/day. [e]Restricted to those who reported being sexually active in past year. [f]Logistic models do not include number of pregnancies.

that use of injectable contraceptives (mainly Depo Provera) for ≥1 year was associated with HSIL/CA (overall RR = 4.2; 95% CI = 0.96–19; RR in analysis restricted to high-risk HPV positives = 6.2; 95% CI = 1.1–34) in a small number of women (3 cases, 8 controls), consistent with previous findings (Herrero et al, 1990). Current IUD users had a RR of 2.9 relative to non-users in analysis restricted to cases with CIN3 or cancer (95% CI = 1.3–6.5).

A> 2-fold association was observed between cigarette smoking and HSIL/CA (Table 2) and risk increased with increasing number of cigarettes smoked per day ($P_{trend}$ = 0.0007). No association was observed between the smoking habits of the husbands/live-in partners of our study participants and risk of HSIL/CA, in analysis restricted to non-smoking women (data not shown).

No significant associations with HSIL/CA were observed for self-reported yeast infections, any of the sexually transmitted diseases (STDs) other than HPV examined (gonorrhoea, syphilis, HSV, other STDs), hand and foot warts, cold sores, other medical conditions (tuberculosis, chicken pox, shingles, asthma), and

 *© 2001 Cancer Research Campaign*

**Table 2** Distribution and risk of HSIL/CA associated with birth control practices and cigarette smoking

| Variable[a] | % Cases (*n* = 146) | % Controls (*n* = 843) | Overall analysis RR[c] | Overall analysis 95% CI | Analysis restricted to women positive for high risk HPV types[b] RR[d] | 95% CI |
|---|---|---|---|---|---|---|
| Oral contraceptive use | | | | | | |
| Never | 36.3 | 34.8 | 1.0 | | 1.0 | |
| Former use | 34.9 | 40.4 | 0.90 | [0.54–1.5] | 0.93 | [0.55–1.6] |
| Current use | 28.8 | 24.8 | 1.6 | [0.89–2.9] | 1.5 | [0.83–2.8] |
| <5 years of use | 37.7 | 44.9 | 0.99 | [0.59–1.7] | 0.99 | [0.58–1.7] |
| 5+ years of use | 26.0 | 20.3 | 1.2 | [0.71–2.2] | 1.3 | [0.70–2.3] |
| Barrier contraceptive use | | | | | | |
| Never | 64.4 | 57.5 | 1.0 | | 1.0 | |
| Former use | 31.5 | 32.1 | 1.0 | [0.65–1.5] | 0.91 | [0.58–1.4] |
| Current use | 4.1 | 10.4 | 0.39 | [0.16–.96] | 0.39 | [0.16–.97] |
| <5 years of use | 25.4 | 32.1 | 0.92 | [0.57–1.5] | 0.85 | [0.52–1.4] |
| 5+ years of use | 2.3 | 10.4 | 0.58 | [0.17–2.1] | 0.40 | [0.09–1.8] |
| | | | $P_{trend}$ = 0.44 | | $P_{trend}$ = 0.22 | |
| Cigarette smoking[e] | | | | | | |
| Never | 79.5 | 90.3 | 1.0 | | 1.0 | |
| Former | 8.2 | 3.7 | 2.4 | [1.2–5.1] | 1.7 | [0.76–4.0] |
| Current | 12.3 | 6.0 | 2.3 | [1.3–4.3] | 2.3 | [1.2–4.3] |
| <10 years of use | 8.2 | 4.9 | 2.6 | [1.2–5.3] | 2.2 | [1.0–4.8] |
| 10 + years of use | 12.3 | 4.8 | 2.2 | [1.2–4.2] | 2.0 | [1.0–3.8] |
| 1–5 cigarettes/day | 15.1 | 7.5 | 2.3 | [1.3–3.9] | 1.8 | [.99–3.3] |
| 6+ cigarettes/day | 5.4 | 2.2 | 2.7 | [1.1–6.7] | 3.1 | [1.2–7.9] |
| | | | $P_{trend}$ = .0007 | | $P_{trend}$ = .003 | |

[a]1 control missing information on oral contraceptive use. 1 control was missing information on ever use and currency of use of barrier contraceptives. 16 cases and 119 controls were missing information on duration of barrier contraceptives. [b]High-risk HPV types include types 16, 18, 31, 33, 35, 39, 45, 51, 52, 56, 58, 59, 68. [c]RR adjusted for age, HPV type, number of pregnancies, and number of cigarettes/day. [d]RR adjusted for age, number of pregnancies, and number of cigarettes/day. [e]RR adjusted for age, HPV types, and number of pregnancies.

**Table 3** RR estimates and 95% confidence intervals for the joint effect of pregnancy and oral contraceptive use[a]

| Oral contraceptive use (# Cases # Controls) | Number of pregnancies (# Cases/# Controls) <3 pregnancies (*n* = 32/372) | 3+Pregnancies (*n* = 114/470) |
|---|---|---|
| Never (53/293) | 1.0 | 4.7 [1.8–12] |
| <5 years (55/378) | 1.8 [.65–4.9] | 3.7 [1.5–9.3] |
| 5+years (38/171) | 3.1 [1.1–9.1] | 4.0 [1.5–10] |

RRs adjusted for age, HPV type, and number of cigarettes/day. [a]Estimates presented are from analysis including all subjects. Similar estimates were obtained in analysis restricted to high-risk HPV positive women.

douching practices (vinegar/water and other preparations) (data not shown).

We estimated the fraction of HSIL/CA that can be attributable to exposures other than HPV among women who are infected with high-risk HPV types. In those women with a high-risk HPV type infection, 42% of HSIL/CA (95% CI = 21–63) can be attributed to multiparity (having ≥3 pregnancies), 10% to cigarette smoking (95% CI = 3–18), and these 2 exposures together account for 44% of HSIL/CA (95% CI = 23–66) (Table 4).

### DISCUSSION

We observed that reproductive practices and smoking are important risk factors for the development of HSIL/CA; in Guanacaste, among women infected with high-risk HPV types, 44% of HSIL/CA can be attributed to these 2 factors. We performed our analysis restricted to only those women with HPV infection and chose controls from a subset of women with HPV infection and without HSIL/CA i.e., LSIL, ASCUS or cytologically normal. We are the first to acknowledge that this may not be an ideal control group. Ideally, one would have wanted to select as controls women who had been infected with HPV in the past (at the same time that the cases were infected) but who resolved their infection (in contrast to the cases, whose infection progressed). Given the lack of a reliable method for the detection of past infection with one of many HPV types, selection of such an ‘ideal’ control group was not possible. In selecting HPV-infected women as controls, we likely biased our control group towards women who have newly acquired HPV infections in which there has been insufficient time for progression to HSIL/CA, or women who have persistent (not transient) HPV infections. Women who have newly acquired infections are more likely to have undergone a change in sexual behaviour that has led to infection. It is unclear whether the inclusion of women with newly acquired infections would attenuate or exaggerate our risk estimates. Persistent HPV infection, which occurs relatively infrequently, is an important precursor to high-grade lesions. Inclusion of these women as controls may therefore have attenuated our findings. This bias, however, could not have explained our positive findings with smoking and reproductive practices. Also reassuring is the fact that in a parallel analysis in which all non-case cohort members were included as controls and adjusting for HPV was achieved through statistical means, identical results were obtained. This suggests that the findings presented herein are robust.

© 2001 Cancer Research Campaign 

**Table 4** Proportion of HSIL and cervical cancer cases attributable to multiparity and cigarette smoking, among women exposed to high-risk HPV

| Exposure | % Exposed | RR[a] | Attributable fraction (95% CI) |
|---|---|---|---|
| >3 pregnancies | 54.7% | 2.2 | 42% (21–63) |
| Cigarette smoking | 10.4% | 2.1 | 10% (3–18) |
| ≥ 3 pregnancies and/or cigarette smoking | 59.3% | 2.2 | 44% (23–66) |

[a]RRs adjusted for age. Pregnancy RR additionally adjusted for number of cigarettes/day. Cigarette smoking RR additionally adjusted for number of pregnancies.

The major strengths of our study are its population-based design and high participation rate (>93%), and its ability to adequately account for confounding by HPV, a known causative agent in the pathogenesis of HSIL/CA. A further strength of the present study was our ability to examine associations restricted to HPV types linked with HSIL/CA, providing further assurance that observations are not confounded by HPV type. Estimates from our analysis restricted to HPV-infected women were identical to those in the population-based analysis, suggesting that HPV was not a significant confounder of the observed associations with high-grade lesions. Our findings substantiate earlier reports that smoking (Reeves et al, 1987; Bosch et al, 1992; Munoz et al, 1993; Ho et al, 1998; Olsen et al, 1998; Roteli-Martins et al, 1998) and parity (Brinton et al, 1980; Bosch et al, 1992; Munoz et al, 1993; Schiffman et al, 1993) were associated with cervical cancer. Finally, since the Pap smear screening behaviour of cases and controls were similar in our rural population, the observed effects cannot be explained by differential detection due to differences in Pap smear screening.

Among HPV-infected women, no residual effect of lifetime number of sexual partners was observed. This finding, along with the lack of disease association observed for self-reported STDs other than HPV, suggests that STDs other than HPV are unlikely to be important in the progression of HPV to HSIL/CA. Self-reporting of STDs in this population was lower than the expected prevalence for these infections suggesting that there was significant underreporting. To better address the role of non-HPV STDs and lower reproductive tract alterations as risk factors for infected women to progress to high-grade lesions, future studies will use biological assays, such as DNA and serological assays, to ascertain exposure to other STDs, bacterial vaginosis and cervical inflammation. Thus, the unreliability of self-reported STDs will be avoided. The tendency for women with multiple recent partners to be at a non-significantly reduced risk of HSIL/CA is consistent with the natural history of cervical neoplasia, where detectable infections and LSILs occur proximal to exposure (i.e. recent sexual behaviour is closely linked to HPV infection and LSIL) while HSILs do not develop until several years after initial exposure.

The underlying mechanism for the observed association between pregnancies and HSIL/CA is unknown. Our finding that still-births did not increase risk and that caesarean sections did not reduce risk argues against the possibility that trauma during delivery explains the observed associations. Other possible explanations include influences of endogenous hormones, nutrition and modulation of the immune response to HPV by pregnancy (Fife et al, 1987; Schneider et al, 1987; Pater et al, 1988; Mitrani-Rosenbaum et al, 1989; Rando et al, 1989; Smith et al, 1991; Mittal et al, 1993; Arbeit et al, 1996; Bartholomew et al, 1998; Fife et al, 1999). Multiparity was not associated with number of lifetime or recent sexual partners and thus the association with HSIL/CA was not confounded by these sexual parameters.

Various mechanisms might explain the association between smoking and HSIL/CA. Constituents of cigarettes, including carcinogens, have been found in the cervical mucus of female smokers (Prokopczyk et al, 1997). In addition, exposure to tobacco may have a detrimental effect on the ability of the host to mount an effective immune response against viral infections (Johnson et al, 1990; Geng et al, 1996). It is important to point out that the prevalence of smoking among women in our study was low and that among smokers, intensity of use was moderate. In populations where prevalence and intensity of smoking are higher, the effect of smoking on risk of HSIL/CA might be even more dramatic.

Results of studies examining the association between oral contraceptives and HSIL/CA have been conflicting (Bosch et al, 1992; Munoz et al, 1993; Becker et al, 1994; Moreno et al, 1995; Kjaer et al, 1996; Chaouki et al, 1998; Chichareon et al, 1998; Ho et al, 1998; Kruger-Kjaer et al, 1998; Ngelangel et al, 1998; Roteli-Martins et al, 1998). We failed to observe an effect of oral contraceptives overall, but noted a significant association between oral contraceptive use and HSIL/CA among women with <3 pregnancies, an effect also observed with other subdivisions of pregnancy numbers (e.g., <2 pregnancies and <4 pregnancies). While one must be cautious when interpreting results from this subgroup analysis, it is possible that multiparous women who use oral contraceptives are non-compliant or that misclassification of duration of use is higher among multiparous women who start and stop use on multiple occasions. If so, the true effect of oral contraceptives on disease risk might be larger than that observed in our study.

It has been postulated that early initiation of sexual activity is a risk factor for HSIL/CA because of metaplasia resultant from ectopy at young ages (Harris et al, 1980). The lack of association between age at first intercourse, age at first pregnancy, or years between menarche and sexual debut and HSIL/CA in our study argues against this ‘vulnerability period’ theory.

It is unclear why barrier contraception (condoms and/or diaphragms) was associated with reduced risk of HSIL/CA among already HPV-infected women. It is possible that barrier users vary from nonusers with regards to unmeasured lifestyle factors associated with disease progression, or that barrier contraceptive use modulates the degree of exposure to HPV. The protective effect observed among barrier contraceptive users might also reflect a protection afforded by barrier contraceptives against STDs other than HPV. It should be noted, however, that no effect of self-reported STDs on risk of HSIL/CA was observed in our study, as discussed above. Replication of this unexpected finding is required before it is accepted.

 *© 2001 Cancer Research Campaign*

We included as controls HPV-infected women with and without cytological evidence of LSIL. This was done because previous work has shown that LSIL is the cytological manifestation of HPV infection (Schiffman and Brinton, 1995). The fact that HPV-infected women with and without LSIL were similar with respect to most factors examined further justifies combining them into a single group. However, a few differences noted between these 2 groups warrant mention. First, compared to HPV-positive women with no evidence of LSIL, those diagnosed with LSIL were twice as likely to be infected with high-risk HPV types, as noted by us in a previous publication (Herrero et al, 2000). Second, HPV-positive women with LSIL were younger than those without LSIL suggesting that in older women HPV infections are less likely to manifest themselves cytologically.

Our study is cross-sectional; replication of our findings in prospectively followed women is needed. Follow-up of women in our Costa Rican cohort should permit such an analysis once sufficient follow-up time is accumulated. In the prospective study, we can use more appropriate controls, women who get an HPV infection at the same time as cases but instead of progressing to high-grade lesions, they resolved their infection. Another limitation of our study is the availability of sensitive PCR-based HPV testing for only a subset of participants. Some cytologically normal women with low-level HPV infections could have been missed by our testing strategy. If women with low-level infections differ from those with higher level infections with respect to the risk factors we examined, our risk estimates could be biased. This bias, however, would likely have attenuated differences between our cases and controls and should not explain away any of our findings. Our study had limited power to evaluate CIN2 cases separately from CIN3/cancer cases. However, our findings are robust since exclusion of CIN2 cases did not alter results. Another limitation is the relatively small number of cancer cases, which precluded analyses to distinguish factors associated with HSIL from those associated with the transition from HSIL to invasive cancer. Since HSIL is the immediate precursor of cervical cancer and since the absolute risk of HSIL progression without treatment is high, factors linked to HSIL are also likely to be linked to invasive cancer. In fact, excluding the cancer cases and restricting our analysis to HSIL alone did not alter our risk estimates. However, it is likely that our study would have missed factors uniquely associated with the progression of HSIL to cervical cancer.

In summary, we observed in our population-based study restricted to HPV positive women that reproductive practices and cigarette smoking are important co-factors for the development of HSIL/CA. Barrier contraceptive use may reduce the risk of HSIL/CA while OC use may be associated with an increased risk of disease in a subgroup of women.

### ACKNOWLEDGEMENTS

This project was supported by a series of National Cancer Institute contracts. We wish to acknowledge the collaboration of Julie Buckland (IMS, Rockville, MD) for data management. We thank Deidra Kelly (Johns Hopkins University) and Dr Laurie Mango (Neuromedical Systems, NY) for their collaboration in the interpretation of cytological specimens. Reagents and services were supplied or discounted by Cytyc Inc (Boxborough, MA), National Testing Laboratories (Fenton, MO), Utah Medical (Midvale, UT), and Neuromedical Systems (Suffern, NY). We offer special recognition for the excellent work of the study staff in Costa Rica, in particular Fernando Cardenas, Manuel Barrantes, Elmer Perez, Lidia Ana Morera and Iris Ugarte. We also acknowledge the collaboration of health authorities in Costa Rica for their enthusiastic support of this project and of the outreach workers of the Ministry of Health of Costa Rica who carried out the population census for their dedication to the health of the people of Guanacaste. We thank Dr Sophia Wang (NCI) for her critical review of this manuscript. Dr Lorincz is scientific director of Digene and holds Digene stock and stock options.

### REFERENCES

Arbeit JM, Howley PM and Hanahan D (1996) Chronic estrogen-induced cervical and vaginal squamous carcinogenesis in human papillomavirus type 16 transgenic mice. *Proc Natl Acad Sci* **93**: 2930–2935

Bartholomew JS, Glenville S, Sarkar S, Burt DJ, Stanley MA, Ruiz-Cabello F, Chengang J, Garrido F and Stern PL (1998) Integration of high risk human papillomavirus DNA is linked to the down-regulation of class I human leucocyte antigens by steroid hormones in cervical tumour cells. *Cancer Res* **57**: 937–942

Becker TM, Wheeler CM, McGough NS, Stidley CA, Parmenter CA, Dorin MH and Jordan SW (1994) Contraceptive and reproductive risks for cervical dysplasia in southwestern hispanic and non-hispanic white women. *Int J Epidemiol* **23**: 913–922

Benichou GJ (2000) Variance calculations and confidence intervals for estimates of the attributable risk based on logistic models. *Biometrics* **46**: 991–1003

Bosch FX, Munoz N, De Sanjose S, izarzugaza I, Gili M, Viladiu P, Tormo MJ, Moreo P, Ascunce N, Gonzalez LC, Tafur L, Kaldor JM, Guerrero E, Aristizabal N, Santamaria M, Alonso de Ruiz P and Shah K (1992) Risk factors for cervical cancer in Colombia and Spain. *Int J Cancer* **52**: 750–758

Bosch FX, Manos MM, Munoz N, Sherman ME, Jansen A, Peto J, Schiffman MH, Moreno V and Shah KV, the IBSCC study group (1995) Prevalence of HPV DNA in cervical cancer: a worldwide perspective. *J Natl Cancer Inst* **87**: 796–802

Breslow NE and Day NE (1980) Statistical methods in cancer research: the analysis of case–control studies. IARC Publ

Brinton LA, Reeves WC, Brenes MM, Herrero R, De Britton RC, Gaitan E, Tenorio F, Garcia M and Rawls WE (1980) Parity as a risk factor for cervical cancer. *Am J Epidemiol* **130**: 486–495

Chaouki N, Bosch FX, Munoz N, Meijer CJLM, El Gueddari B, El Ghazi A, Deacon J, Castellsague X and Walboomers J (1998) The viral origin of cervical cancer in Rabat, Morocco. *Int J Cancer* **75**: 546–554

Chichareon S, Herrero R, Munoz N, Bosch FX, Jacobs MV, Deacon J, Santamaria M, Chongsuvivatwong V., Meijer CJLM and Walboomers JMM (1998) Risk factors for cervical cancer in Thailand: a case-control study. *J Natl Cancer Inst* **90**: 50–57

Cox JT, Lorincz AT, Schiffman MH, Sherman ME, Kurman RJ, Hussein M and Cullen A (1995) Human papillomavirus testing by Hybrid Capture appears to be useful in triaging women with a cytologic diagnosis of atypical squamous cells of undetermined significance. *Am J Obstet Gynecol* **172**: 946–954

Fife KH, Rogers RE and Zwickl BW (1987) Symptomatic and asymptomatic cervical infections with human papillomavirus during pregnancy. *J Infect Dis* **156**: 904–911

Fife KH, Katz BP, Brizendine EJ and Brown DR (1999) Cervical human papillomavirus deoxyribonucleic acid persists throughout pregnancy and decreases in the postpartum period. *Am J Obstet Gynecol* **180**: 1110–1114

Geng Y, Savage SM, Razanai-Boroujerdi S and Sopori ML (1996) Effects of nicotine on the immune response. II. Chronic nicotine treatment induces T cell anergy. *J Immunol* **156**: 2384–2390

Harris RWC, Brinton LA, Cowdell RH, Skegg DCG, Smith PG, Vessey MP and Doll R (1980) Characteristics of women with dysplasia or carcinoma in situ of the cervix uteri *Br J Cancer* **42**: 359–369

Herrero R, Brinton LA, Reeves WC, Brenes MM, de Britton RC, Tenorio F and Gaitan E (1990) Injectable contraceptives and risk of invasive cervical cancer: evidence of an association. *Int J Cancer* **46**: 5–7

Herrero R, Schiffman MH, Bratti C, Hildesheim A, Balmaceda I, Sherman ME, Greenberg M, Cardenas F, Gomez V, Helgesen K, Morales J, Hutchinson M, Mango L, Alfaro M, Potischman NW, Wacholder S, Swanson C and Brinton LA (1997) Design and methods of a population-based natural history study of cervical neoplasia in a rural province of Costa Rica: the Guanacaste project. *Pan Am J Public Health* **1**: 362–374

© 2001 Cancer Research Campaign

Herrero R, Hildesheim A, Bratti C, Sherman ME, Hutchinson M, Morales J, Balmaceda I, Greenberg MD, Alfaro M, Burk RD, Wacholder S and Schiffman M (2000) A population-based study of human papillomavirus infection and all grades of cervical neoplasia in rural Costa Rica. *J Natl Cancer Inst* **92**: 464–474

Hildesheim A, Schiffman MH, Gravitt P, Glass AG, Greer C, Zhang T, Scott DR, Rush BB, Lawler P, Sherman ME, Kurman RJ and Manos MM (1994) Persistence of type-specific human papillomavirus infection among cytologically normal women in Portland, Oregon. *J Infect Dis* **169**: 235–240

Ho GY, Kadish AS, Burk RD, Basu J, Palan PR, Mikhail MS and Romney SL (1998) HPV 16 and cigarette smoking as risk factors for high-grade cervical intra-epithelial neoplasia. *Int J Cancer* **78**: 281–285

Johnson JD, Houchens DP, Kluwe WM, Craig DK and Fisher GL (1990) Effects of mainstream and environmental tobacco smoke on the immune system in animals and humans: a review. *Toxicology* **20**: 369–395

Kjaer SK, van den Brule AJC, Bock JE, Poll PA, Engholm G, Sherman ME, Walboomers JMM and Meijer CJLM (1996) Human papillomavirus-The most significant risk determinant of cervical intraepithelial neoplasia. *Int J Cancer* **65**: 601–606

Kruger-Kjaer S, Van den Brande JL, Svare E, Engholm G, Sherman ME, Poll PA, Walboomers JMM, Bock JE and Meijer CJLM (1998) Different risk factor patterns for high-grade and low-grade intraepithelial lesions of the cervix among HPV-positive and HPV-negative young women. *Int J Cancer* **76**: 613–619

Mitrani-Rosenbaum S, Tsvieli R and Tur-Kaspa R (1989) Oestrogen stimulates differential transcription of human papillomavirus type 16 in SiHa cervical carcinoma cells. *J gen Virol* **70**: 2227–2232

Mittal R, Tsutsumi K, Pater A and Pater MM (1993) Human papillomavirus type 16 expression in cervical keratinocytes: role of progesterone and glucocorticoid hormones. *Obstet Gynecol* **81**: 5–12

Moreno V, Munoz N, Bosch FX, De Sanjose S, Gonzalez LC, Tafur L, Gili M, Izarzugaz I, Navarro C, Vergara A, Viladiu P, Ascunce N and Shah F (1995) Risk factors for progression of cervical intraepithelial neoplasm grade III to invasive cervical cancer. *Cancer Epidemiol Biomarkers Prev* **4**: 459–467

Munoz N, Bosch FX, deSanjose S, Vergara A, delMora A, Munoz MT, Tafur L, Gili M, izarzugaza I, Viladiu P, Navarro C, Alonso de Ruiz P, Aristizabal N, Santamaria M, Orfila J, Daniel RW, Guerrero E and Shah KV (1993) Risk factors for cervical intraepithelial neoplasia grade III/carcinoma in situ in Spain and Colombia. *Cancer Epidemiol Biomarkers Prev* **2**: 423–431

Ngelangel C, Munoz N, Bosch FX, Limson GM, Festin MR, Deacon J, Jacobs MV, Santamaria M, Meijer CJLM and Walboomers JMM (1998) Causes of cervical cancer in the Philippines: a case-control study. *J Natl Cancer Inst* **90**: 43–49

Nobbenhuis MAE, Walboomers JMM, Helmerhorst TJM, Rozendaal L, Remmink AJ, Risse EKJ, van der Linden HC, Voorhorst FJ, Kenemans P and Meijer CJL (1999) Relation of human papillomavirus status to cervical lesions and consequences for cervical-cancer screening: a prospective study. *The Lancet* **354**: 20–25

Olsen AO, Dillner J, Skrondal A and Magnus P (1998) Combined effect of smoking and human papillomavirus type 16 infection in cervical carcinogenesis. *Epidemiology* **9**: 346–349

Pater MM, Hughes GA, Hyslop DE, Nakshatri H and Pater A (1988) Glucocorticoid-dependent oncogenic transformation by type 16 but not type 11 human papilloma virus DNA. *Nature* **27**: 832–835

Prokopczyk B, Cox JE, Hoffmann D and Waggoner SE (1997) Identification of tobacco-specific carcinogen in the cervical mucus of smokers and nonsmokers. *J Natl Cancer Inst* **89**: 868–873

Rando RF, Lindheim S, Hasty L, Sedlacek TV, Woodland M and Eder C (1989) Increased frequency of detection of human papillomavirus deoxtribonucleic acid in exfoliated cervical cells during pregnancy. *Am J Obstet Gynecol* **161**: 50–55

Reeves WC, Caussy D, Brinton LA, Brenes MM, Montalvan P, Gomez B, De Brinton RC, Morice E, Gaitan E, De Lao SL and Rawls WE (1987) Case-control study of human papillomaviruses and cervical cancer in Latin America. *Int J Cancer* **40**: 450–454

Roteli-Martins CM, Panetta K, Alves VAF, Siqueira SAC, Syrjanen KJ and Derchain SFM (1998) Cigarete smoking and high-risk HPV DNA as predisposing factors for high-grade cervical intraepithelial neoplasia (CIN) in young Brazilian women. *Acta Obstet Gynecol Scand* **77**: 678–682

Schiffman MH and Brinton LA (1995) The epidemiology of cervical carcinogenesis. *Cancer* **76**: 1888–1901

Schiffman MH, Bauer HM, Hoover RN, Glass AG, Cadell DM, Rush BB, Scott DR, Sherman ME, Kurman RJ, Wacholder S, Stanton CK and Manos MM (1993) Epidemiologic evidence showing that human papillomavirus infection causes most cervical intraepithelial neoplasia. *J Natl Cancer Inst* **85**: 958–964

Schiffman MH, Brinton LA, Devesa SS and Fraumeni JF, Jr. (1996) Cervical cancer. In: *Cancer epidemiology and prevention*, Schottenfeld D and Fraumeni JF, Jr. (eds) pp 1090–1116. Oxford Univ Press: New York

Schiffman M, Herrero R, Hildesheim A, Sherman ME, Bratti MC, Wacholder S, Alfaro M, Hutchinson M, Morales J, Greenberg MD and Lorincz AT (1999) HPV DNA testing in cervical cancer screening: results from women in a high-risk province of Costa Rica. *JAMA* **283**: 87–93

Schneider A, Hotz M and Gissmann L (1987) Increased prevalence of human papillomavirus in the lower genital tract of pregnant women. *Int J Cancer* **40**: 198–201

Smith EM, Johnson SR, Jiang D, Zaleski S, Lynch CF, Brundage S, Anderson RD and Turek LP (1991) The association between pregnancy and human papilloma virus prevalence. *Cancer Detect Prev* **15**: 397–402

 *© 2001 Cancer Research Campaign*
